# Experimental learning of quantum states

**DOI:** 10.1126/sciadv.aau1946

**Published:** 2019-03-29

**Authors:** Andrea Rocchetto, Scott Aaronson, Simone Severini, Gonzalo Carvacho, Davide Poderini, Iris Agresti, Marco Bentivegna, Fabio Sciarrino

**Affiliations:** 1 Department of Computer Science, University of Oxford, Oxford, UK.; 2 Department of Computer Science, University College London, London, UK.; 3 Department of Computer Science, University of Texas at Austin, Austin, USA.; 4 Institute of Natural Sciences, Shanghai Jiao Tong University, Shanghai, China.; 5 Dipartimento di Fisica–Sapienza Università di Roma, Rome, Italy.

## Abstract

The number of parameters describing a quantum state is well known to grow exponentially with the number of particles. This scaling limits our ability to characterize and simulate the evolution of arbitrary states to systems, with no more than a few qubits. However, from a computational learning theory perspective, it can be shown that quantum states can be approximately learned using a number of measurements growing linearly with the number of qubits. Here, we experimentally demonstrate this linear scaling in optical systems with up to 6 qubits. Our results highlight the power of the computational learning theory to investigate quantum information, provide the first experimental demonstration that quantum states can be “probably approximately learned” with access to a number of copies of the state that scales linearly with the number of qubits, and pave the way to probing quantum states at new, larger scales.

## Teaser

A photonic system is used to demonstrate that quantum states can be approximately learned using a linear number of measurements.

## INTRODUCTION

The exponential scaling of the wave function, arising from the tensor product description of multiparticle states, is one of the remarkable properties of quantum systems, and if exploited correctly, it is instrumental in powering the computational advantages theorized in quantum information processing. On the other hand, an exponentially increasing computational space makes the evolution of quantum systems hard to simulate with classical methods. For example, calculating a single amplitude exactly is known to be a #P-hard problem (*1*), where #P can be viewed as the counting equivalent of NP.

Some of the limitations set by the exponential scaling of the wave function can be formalized in quantum-state tomography (*2*–*8*). The central task of quantum-state tomography is to produce a description of an *n*-qubit state given the ability to prepare and measure *k* of its copies. Characterizing an unknown quantum state is a fundamental tool in quantum information processing. A survey of the major applications and present challenges in state tomography can be found in the review by Banaszek *et al*. (*2*). State estimation is, in general, an expensive procedure. For an arbitrary *n*-qubit quantum state, it can be shown that estimating the ideal state up to an approximation parameter ε requires Ω(4^*n*^/ε^2^) operations (*3*). Prior information plays an important role in any procedure seeking to characterize a quantum state. In quantum-state tomography, for example, knowing that the state is low rank (*4*, *8*), or that it has a matrix product state structure (*7*), can reduce the computational cost of the procedure to polynomial in the number of qubits. More generally, self-testing (*9*), a type of device-independent state characterization, is possible only for specific class of states, such as multipartite qubit states that admit a Schmidt decomposition (*10*), whose structure is known a priori. Despite the existence of these efficient protocols, there is no hope of overcoming the exponential scaling for general unknown quantum states. Given this difficulty, it is valuable to interpret quantum-state tomography as a learning problem, with the hope of using the well-developed machinery of computational learning theory, for optimizing the number of required measurements.

Computational learning theory (*11*, *12*) is a research field devoted to studying the design and analysis of machine learning algorithms. Particularly relevant for our purposes is supervised machine learning. Here, the learner is presented with a number of examples consisting of input-output pairs and is subsequently assigned the task of predicting the output of a new input. This model of learning has been formalized in computational learning theory by Valiant in 1984 (*13*) with the introduction of the probably approximately correct (PAC) model. A defining feature of this setting is that the accuracy of the learner is measured under the same probability distribution that models the training set. The PAC framework provides two indicators of the efficiency of a learner: the sample complexity and the time complexity. The first is the worst-case number of examples it uses to reach some target competency, while the second one is the worst-case running time of the learner. In this article, we are concerned with the sample complexity of the problem of learning quantum states.

Quantum-state tomography can be cast as a learning problem. In this perspective, the learner makes use of the training set to produce a hypothesis that can predict any measurement on the state. Here lies a crucial difference with the setting defined in the PAC model where the learner can predict, and with a nonzero failure probability, only measurements that are similar to those seen in the training set. Since quantum-state tomography requires an exponentially large number of measurements, we might conclude that the same applies to the problem of PAC learning quantum states.

Computational learning theory, and in particular the PAC model, can help to address these conundrums. By analyzing quantum-state tomography from a computational learning perspective, Aaronson (*14*) proved that quantum states can be PAC-learned with a linearly scaling training set. Note that quantum-state tomography is inherently different from the PAC framework discussed in this paper. While in the first setting, the task is to predict the outcome probabilities of any measurement performed on the state, in the latter, the learner is only required to predict measurements sampled from an unknown probability distribution. Within the boundaries of this precise definition of learning, it is possible to think of quantum states as having linear sample complexity. Here, we present the first experimental demonstration of such linear scaling. Our contributions also include developing a testable model for the main theorem proved in (*14*) and estimating an important scaling constant. We run the experiments on a photonic platform including up to 6 qubits. Our results experimentally demonstrate an important property of quantum states and highlight the power of computational learning theory in the quantum information framework.

## RESULTS

### Quantum learnability theory

Let us recall some standard definitions in quantum theory. A generic *n*-qubit state ρ is a trace-one, positive semidefinite matrix acting on a Hilbert space of dimension 2^*n*^. Every observation of a state is mathematically described by a positive operator valued measurement (POVM), *E* = {*E*^(*j*)^}, where each *E*^(*j*)^ is a Hermitian-positive semidefinite operator such that ∑_*j*_*E*^(*j*)^ = *I*. The probability of measurement outcome *j* is *p*(*j*) =Tr(*E*^(*j*)^ρ). For our purposes, we refer to a measurement of ρ as a two-outcome POVM {*E*^(1)^ = *E*, *E*^(2)^ = 1 − *E*} with eigenvalues in [0, 1] (notice that the results presented below can be extended to the case of *k*-outcome POVMs). We denote by S the set of all measurements on *n* qubits.

Following (*14*), we define the learning of ρ as the task of processing a training set composed of *m* tuples {(*E*_*i*_, Tr(*E*_*i*_ρ))}, drawn from a probability distribution D, to predict the “behavior” of ρ on most measurements drawn from D. This concept of learning is defined in the context of Valiant’s PAC model (*13*). In this framework, originally developed for Boolean functions but then extended to real-valued ones by Bartlett *et al*. (*15*), a learning algorithm (the learner) tries to approximate with a high probability an unknown function f:X→Y from a training set of random-labeled examples. Each labeled example is of the form (*x*, *f*(*x*)), where *x* is distributed according to some unknown distribution D. To make learning possible, we restrict the hypothesis that the learner can use to approximate *f* to a set of functions H={h:X→Y}. We refer to H as the hypothesis class. The learning algorithm takes as input the training set and generates a hypothesis *h* ∈ H that approximates *f*. The PAC model makes use of two approximation parameters, ε and δ. The accuracy parameter ε determines how far the hypothesis *h* can be from *f*. The confidence parameter gives the probability of sampling a training set that is not representative of the underlying distribution D. A hypothesis class H is said to be PAC learnable if there exists an algorithm that, for every probability distribution D and function *f* and for every ε, δ ∈ (0, 1); when running the learning algorithm on *m* ≥ *m*_H_ examples drawn from D, we have that, with a probability of at least 1 − δ

$$\underset{x\sim D}{\text{Pr}}[h(x)\neq f(x)]\leq \varepsilon$$

Here, by ~, we indicate that *x* is drawn from D. The value *m*_H_ determines the minimum number of examples required to PAC-learn the class H. We refer to *m*_H_ as the sample complexity of the hypothesis class H. We note that the learner must test the predictions under the same distribution D that determines the elements in the training set.

The PAC-model has been adapted to quantum states in (*14*). Here, the learner tries to approximate a function $F_{\rho }:S\to [0,1]$, where *F*_ρ_ is defined as $F_{\rho }(E_{i}^{\left(1\right)})=\;\text{Tr}(E_{i}^{\left(1\right)}\rho )$. The training set corresponds to a set of *m* tuples $\left\{(E_{i}^{\left(1\right)},F_{\rho }(E_{i}^{\left(1\right)}))\right\}$. Notice that we always take the first element $E_{i}^{\left(1\right)}$ of each POVM *E*_*i*_. For this reason, in the following, we take $E_{i}^{\left(1\right)}=E_{i}$. The POVMs {*E*_*i*_} are drawn from an unknown distribution D, and the *F*_ρ_(*E*_*i*_) values are determined experimentally. After processing the training set, the learner outputs an hypothesis state σ. Notice that an efficient learner must output an efficient classical description of the hypothesis. A quantum state is considered to be learned if, with probability 1 − δ, a training set generated according to the distribution D can be used to predict with probability ε and accuracy γ any other measurement drawn from D

$$\underset{E\in D}{\text{Pr}}[|\text{Tr}(E\sigma )-\text{Tr}(E\rho )|>\gamma ]\leq \varepsilon$$ (1)

**Fig. 1:**
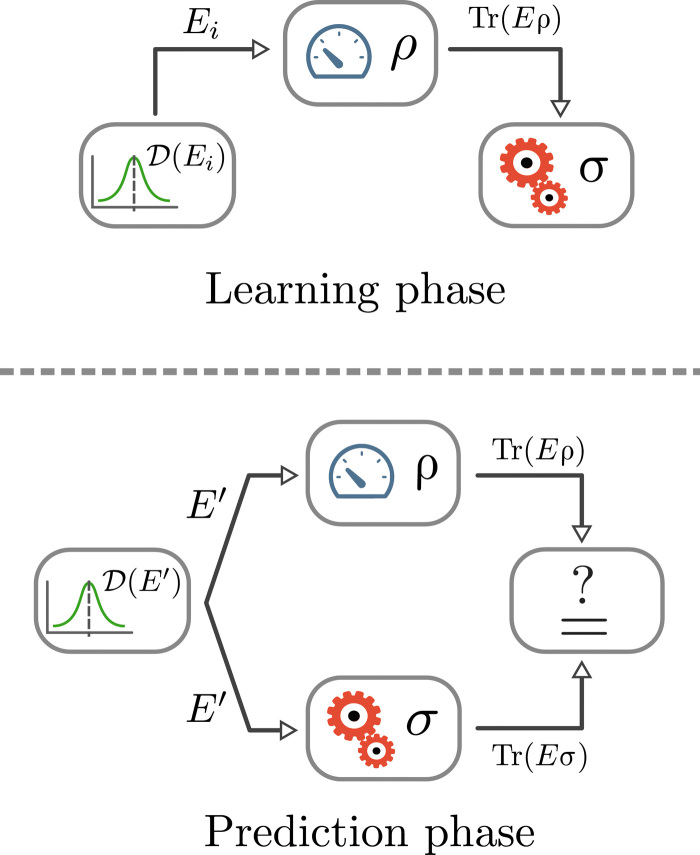
Schematic of the learning procedure. (**Top**) In the learning phase, measurements drawn randomly from D are performed on the physical state ρ. On the basis of the measurement outcomes, the learning algorithm outputs a hypothesis σ. (**Bottom**) In the prediction phase, the goal is to predict the experimental outcome of a measurement *E*′ drawn from D using σ as hypothesis.

A pictorial description of this learning procedure is shown in Fig. 1. Because σ is a 2^*n*^ × 2^*n*^-dimensional matrix, we would expect that the number of examples in the training set required to learn ρ also scales exponentially. However, it has been proved (*14*) that the number of examples required to learn *F*_ρ_ scales linearly with *n* and is polynomially inverse with the relevant error parameters (a full statement of the theorem is given in Materials and Methods; in the following, we shall refer to theorem as Theorem 1). More specifically, keeping the error parameters ε, γ, and δ fixed, we can PAC learn a quantum state provided

$$m\geq \frac{K}{\gamma^{4}\varepsilon^{2}}(\frac{n}{\gamma^{4}\varepsilon^{2}}{\text{log}}^{2}\frac{1}{\gamma \varepsilon }+\text{log}\frac{1}{\delta })$$ (2)

where *K* is a constant. This result provides an upper bound on the number of measurements required to learn a quantum state with respect to any probability measure over two-outcome POVMs. The value of *K* is left unbounded, but it is critical for applying the theorem in an experimental setting.

The learning procedure prescribed by Theorem 1 is simple and it involves finding a hypothesis state σ such that Tr(*E*_*i*_σ) ≈ Tr(*E*_*i*_ρ) for all *i*. Then, with high probability, that hypothesis will generalize in the sense that Tr(*E*σ) ≈ Tr(*E*ρ) for most *E*’s drawn from D. It is then possible to interpret the problem of finding a mixed *n*-qubit state that approximately agrees with the measurements as an optimization problem.

The optimization problem takes as input *m* POVMs described by Hermitian matrices {*E*_1_, …, *E*_*m*_} and their corresponding measurement outcomes {Tr(*E*_1_ρ), …, Tr(*E*_*m*_ρ)}. The goal is to find a Hermitian-positive semidefinite matrix σ that minimizes

$$f(\sigma )=\sum_{i=1}^{m}{\left(\text{Tr}(E_{i}\sigma )-\text{Tr}(E_{i}\rho )\right)}^{2}$$ (3)

σ≥0,Tr(σ)=1

where, by σ ≥ 0, we denote the positive semidefiniteness of σ.

The above formulation is a convex program whose solution is known to be computable in polynomial time in the dimension of σ using interior point methods (*16*, *17*) or the ellipsoid method (*18*). However, because the dimension of σ scales exponentially with *n*, the problem of finding the minimum of *f*(σ) is, in practice, not efficiently computable. This is still compatible with the linear scaling of Theorem 1 (see Materials and Methods) because the results proved in (*14*) are purely information theoretic and are concerned only with the sample size *m*. For any given class of quantum states, the question of whether hypothesis states can be produced efficiently is still open. In this context, Rocchetto (*19*) recently proved that stabilizer states are efficiently PAC learnable.

Last, we note that learning a quantum state is not a complete replacement for standard quantum-state tomography. The PAC-learning framework of Theorem 1 tests the predictions over the same distribution of the training set; a good hypothesis state could be arbitrarily far from the true state in the usual trace distance metric, but hard to distinguish from the true state with respect to the given distribution over measurements.

### Experimental setup

**Fig. 2:**
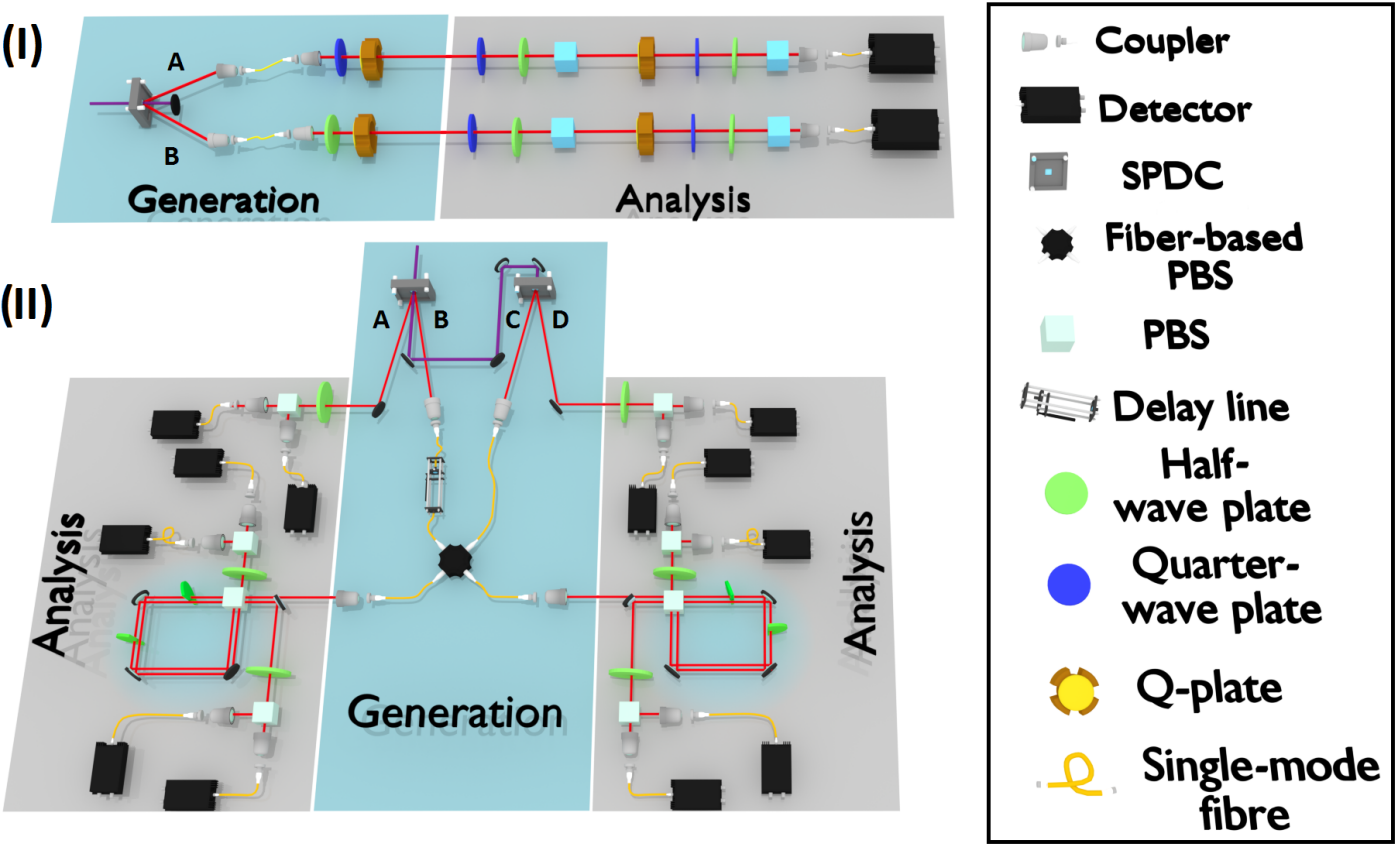
Experimental setups for generating the 3-, 4-, 5-, and 6-qubit GHZ states. Pictorial representation of the two different experimental setups used to generate the quantum states learned with Theorem 1. In setup (I), we make use of two photons and encode up to 4 qubits. In setup (II), we make use of four photons and encode up to 6 qubits. (**I**) In the generation stage, the state of each of the two entangled photons (1 and 2) is locally manipulated via QWPs, HWPs, and q-plates, set to generate a specific GHZ state. The analysis is performed using QWPs, HWPs, and polarizing beam splitters (PBSs). The orbital angular momentum (OAM) analysis requires a q-plate to transfer the information encoded in the OAM space to the polarization degree of freedom, which can be then analyzed with standard techniques. After the analysis, both photons are sent to single-mode fibers (SMFs) connected to single-photon detectors. (**II**) Two polarization-entangled photon pairs are generated via SPDC in two separated nonlinear crystals. Photons A and D of the first and second pair, respectively, are sent directly to a HWP and a PBS for polarization analysis. Photons B and C instead are sent to a 50/50 in-fiber PBS followed by another PBS, which realizes the polarization-path entanglement. The two paths go through two HWPs and are rejoined in the same PBS, forming a Sagnac-like configuration, whose main role is to guarantee phase stability and perform path-polarization measurements independently. A motorized delay line is adopted to change the photons wave-packet temporal overlap in the PBS. Each path analysis section is composed of a HWP and a PBS after which the photons are coupled into SMFs connected to single-photon detectors. Generation and analysis sections are represented by cyan and gray zones, respectively.

We test the learning Theorem 1 over different Greenberger-Horne-Zeilinger (GHZ) states (*20*) (see Materials and Methods for a definition). There are several methods to produce GHZ states (*21*–*25*) in photonic systems. To scale up to 6 qubits, we use two different approaches: The first one aims to increase the number of degrees of freedom per photon, while the second one exploits an increasing number of photons (see Fig. 2). In setup (I), we generate two-photon states, encoding up to 4 qubits, and perform a full set of measurements in the computational basis. In setup (II), we generate four-photon states, able to encode up to 6 qubits. Both setups exploit spontaneous parametric down-conversion (SPDC) to generate polarization-entangled photons pairs (see Materials and Methods).

In setup (I), depicted in Fig. 2, we use the q-plate (*26*), a birefringent-patterned slab, to entangle polarization and orbital angular momentum (OAM) of single photons (*27*–*30*). This makes encoding 2 qubits per photon possible, exploiting their polarization and OAM degrees of freedom. To obtain a 4-qubit GHZ state, the q-plate acts on the Bell state $\left|\psi^{-}\rangle =\frac{1}{\sqrt{2}}(|RL\rangle -|LR\rangle \right)$, where *R* and *L* denote the right and left circular polarization of the two photons, respectively, allowing a polarization-controlled variation of the OAM. More specifically, states with right or left polarization become OAM eigenstates with ℓ = −1 or ℓ = +1, respectively. Conditioned on the measurements of a subset of qubits, we can also generate 3- and 2-qubit states, as summarized in Table 1. To perform a complete quantum-state tomography in both Hilbert spaces, the analysis is carried out using two series of quarter-wave plates (QWPs), half-wave plates (HWPs), and polarizing beam splitters (PBSs), separated by another q-plate, to transfer the information from the OAM to the polarization subspace (*30*). The photons are then sent to single-mode fibers (SMFs), which can be coupled only with states carrying null OAM.

**Table 1:** Qubit encoding. The table shows the encoding map between logical states and photons. Photons are labeled with capital letters A, B, C, and D. Two photons (A and B in the table) are used in setup (I) to encode states up to 4 qubits in the polarization and OAM basis. For setup (II), states up to 6 qubits are generated by adding two extra photons (C and D) and using an encoding in path and polarization. The states |*H*〉, |*V*〉, |*R*〉, |*L*〉 denote the polarization degree of freedom, while |+1〉 and |−1〉 represent the eigenstates of the OAM with *l* = +1 and *l* = −1, respectively. To identify the two possible paths of the photons in setup (II), we use the labels |*a*〉 and |*b*〉.

***n* of qubits**	**Experimental** **setup**	**Photon**					
		**A**		**B**		**C**	**D**
2	I	|0〉_1_ = |*H*〉		|0〉_2_ = |*H*〉			
		|1〉_1_ = |*V*〉		|1〉_2_ = |*V*〉			
3	I	|0〉_1_ = |*R*〉	|0〉_2_ = |+1〉	|0〉_3_ = |*R*〉			
		|1〉_1_ = |*L*〉	|1〉_2_ = |−1〉	|1〉_3_ = |*L*〉			
4	I	|0〉_1_ = |*R*〉	|0〉_2_ = |+1〉	|0〉_3_ = |*R*〉	|0〉_4_ = |+1〉		
		|1〉_1_ = |*L*〉	|1〉_2_ = |−1〉	|1〉_3_ = |*L*〉	|1〉_4_ = |−1〉		
3	II	|0〉_1_ = |*H*〉		|0〉_2_ = |*H*〉		|0〉_3_ = |*H*〉	
		|1〉_1_ = |*V*〉		|1〉_2_ = |*V*〉		|1〉_3_ = |*V*〉	
4	II	|0〉_1_ = |*H*〉		|0〉_2_ = |*H*〉		|0〉_3_ = |*H*〉	|0〉_4_ = |*H*〉
		|1〉_1_ = |*V*〉		|1〉_2_ = |*V*〉		|1〉_3_ = |*V*〉	|1〉_4_ = |*V*〉
5	II	|0〉_1_ = |*H*〉	|0〉_2_ = |*a*〉	|0〉_3_ = |*H*〉		|0〉_4_ = |*H*〉	|0〉_5_ = |*H*〉
		|1〉_1_ = |*V*〉	|1〉_2_ = |*b*〉	|1〉_3_ = |*V*〉		|1〉_4_ = |*V*〉	|1〉_5_ = |*V*〉
6	II	|0〉_1_ = |*H*〉	|0〉_2_ = |*a*〉	|0〉_3_ = |*H*〉	|0〉_4_ = |*a*〉	|0〉_5_ = |*H*〉	|0〉_6_ = |*H*〉
		|1〉_1_ = |*V*〉	|1〉_2_ = |*b*〉	|1〉_3_ = |*V*〉	|1〉_4_ = |*b*〉	|1〉_5_ = |*V*〉	|1〉_6_ = |*V*〉

In setup (II), depicted in Fig. 2, we encode the qubits in the polarization and path degrees of freedom. Through this encoding, we can generate four-photon states and up to 6 qubits. This setup involves two separate SPDC sources, which generate two pairs of polarization-entangled photons, (A and B) and (C and D), with the same pulse of the laser. We can then obtain a 4-qubit GHZ state encoded in polarization, by simultaneously injecting one photon from each source (B and C) over the two inputs of a fiber-based PBS. In this configuration, each photon carries 1 qubit. The dimension of the system can be increased to 5 qubits by sending one of the two output modes of the fiber-based PBS in a Sagnac interferometer (shown in Fig. 2). This allows us to entangle and measure the polarization and path degrees of freedom of a single photon while retaining phase stability. This scheme can be easily extended to 6 qubits by sending the other output mode of fiber-based PBS in another Sagnac interferometer. In this case, two out of the four photons carry 2 qubits, which are encoded in the polarization and path degree of freedom, as shown in Table 1. Through the above procedures, we can generate the state

$$\left|{\text{GHZ}}_{n}\rangle =\frac{1}{\sqrt{2}}({|0\rangle }^{⊗n}+{|1\rangle }^{⊗n}\right)$$ (4)

for *n* = 3, 4, 5, 6 qubits. The polarization analysis is performed with a HWP and a PBS for each path.

### Experimental demonstration

We demonstrate numerically and experimentally, through two photonic systems able to encode from 2 to 6 qubits, that quantum states can be PAC-learned with a linearly scaling training set, that is, we demonstrate that the number of elements *m* in the training set required to learn an *n*-qubit quantum state ρ scales linearly with *n*.

Although Theorem 1 can be applied under any distribution D, it is interesting to test its prediction under distributions that make the learning problem challenging. If, for example, one were to take the uniform distribution over all possible measurement bases, then, with a high probability, no measurement drawn from this distribution would be able to distinguish the state from the completely mixed one (the expected value of an exponentially big fraction of the measurements would be equal to 12). We define the completely mixed state as the state described by the density matrix *I*/2^*n*^, where, by *I*, we denote the identity matrix.

All of our experiments are performed on GHZ states, a type of stabilizer state (see Materials and Methods for further details). We remark that, because of experimental noise, we are effectively testing the learnability of a mixed state and not of perfect GHZ states. The advantage of using states that are close to GHZs, a class that is known to admit an efficient representation, is the possibility of identifying a set of measurements and a probability distribution that make the predictions of theorem “interesting” in the sense that they cannot be reproduced using the completely mixed state as hypothesis. Last, we note that our learning algorithm does not exploit the GHZ structure of the states.

Depending on the experimental setup, we use two probability distributions, $D_{\left(I\right)}$ for setup (I) and $D_{\left(\text{II}\right)}$ for setup (II), that are uniform over a subset of the stabilizers of the state (details on the distributions can be found in Materials and Methods). Under these distributions, the completely mixed state is never a good hypothesis (unless γ > 0.5) because the stabilizer measurements performed on the state will always return 1 as an outcome. On the completely mixed state, the same measurements will output 1 or 0 with equal probability.

In the case of learning with experimental data, we have to take into account two factors that can invalidate Theorem 1: noise in the measurements and the lack of access to the true value of Tr(*E*ρ). Both issues can be positively addressed. We examine the noise problem first. As discussed in (*14*), if the noise that corrupts *E* to *E*′ is governed by a known probability distribution such as a Gaussian, then *E*′ is still just a POVM, so Theorem 1 applies directly. If the noise is adversarial, then we can also apply Theorem 1 directly, provided we have an upper bound on $\left|\text{Tr}(E_{i}\rho )-\text{Tr}({E\prime }_{i}\rho )\right|$. As for the second issue, approximate values of the expectation values are also within the validity of the theorem. A discussion is provided in Materials and Methods.

**Fig. 3:**
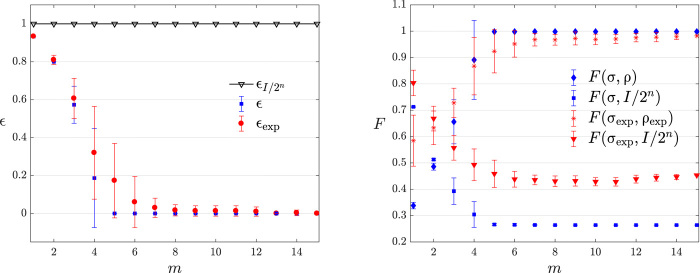
Learning of a 4-qubit GHZ state. Numerical simulations (blue curves) and experimental data (red curves) of the learning of the state $(0000+1111)/\sqrt{2}$. The subscript [⋅]_exp_ denotes experimental data. Quantities without a subscript are obtained through numerical simulations. (**Left**) The probability ε of predicting a measurement outcome with less than γ = 0.1 accuracy. The black line represents the predictions made using the completely mixed state as hypothesis. The informed predictions are always better than a random guess. (**Right**) The fidelity $F=\;\text{Tr}(\sqrt{\sigma^{1/2}\rho \sigma^{1/2}})$ between the hypothesis state σ reconstructed by the PAC-learning algorithm and ρ and between σ and the completely mixed state *I*/2^*n*^, that is, the starting guess of the optimization algorithm. A discussion on the high variance of the data points with *m* = 4 is provided in Materials and Methods. The learning distribution $D_{\left(I\right)}$ is uniform over the set of stabilizer measurements of the state minus the identity matrix (see Materials and Methods). Error bars show the SD for an average of 20 different, randomly generated, training sets.

We begin our experimental analysis with a full characterization of the PAC learnability of a four-qubit GHZ state generated with setup (I). The complete set of measurements available with setup (I) allows us to compare the quality of the hypothesis σ, not only in terms of the learning theorem but also from a tomographic perspective. The results, presented in Fig. 3, show that, by increasing the number of measurements in the training set, the hypothesis σ is getting closer, in terms of fidelity, to the ideal state and to the experimental state (right panel). In the same figure, it is possible to see that the predictions (left panel, red dots) obtained by minimizing *f*(σ) are always better than those obtained by taking the completely mixed state (black line) as hypothesis. This confirms that the distributions we selected are interesting from a learning perspective because it is not possible to make good predictions using random guessing.

**Fig. 4:**
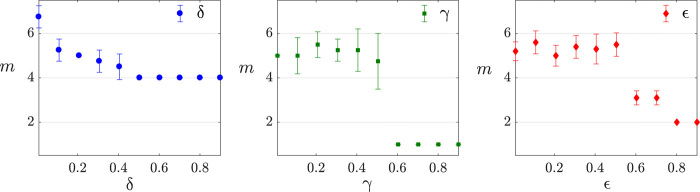
Measurement complexity of error parameters. Dependence of *m* on the error parameters for learning 4-qubit GHZ states generated with setup (I). Learning is performed under the distribution $D_{\left(I\right)}$ (see Materials and Methods for further details), and each data point is an average over four different GHZ states. When a given error parameter is changed, the other ones are kept constant at the following values δ = 0.1, γ = 0.1, and ε = 0.05. (**Left**) Scaling of δ. (**Center**) Scaling of γ. (**Right**) Scaling of ε.

Still, using GHZ states generated from setup (I), we test the dependency of the measurement complexity on the error parameters ε, δ, and γ. This kind of test is necessary to ensure that the hardness of the learning problem used in the experimental demonstration of the theorem is representative of a typical learning scenario. The numerical simulations on the scaling of the error parameters are shown in Fig. 4 and indicate that, as expected from Eq. 2, the hardness of the learning problem does not change abruptly with the error parameters (unless they introduce pathological cases; for example, for γ > 0.5, random guessing becomes a good prediction strategy).

**Fig. 5:**
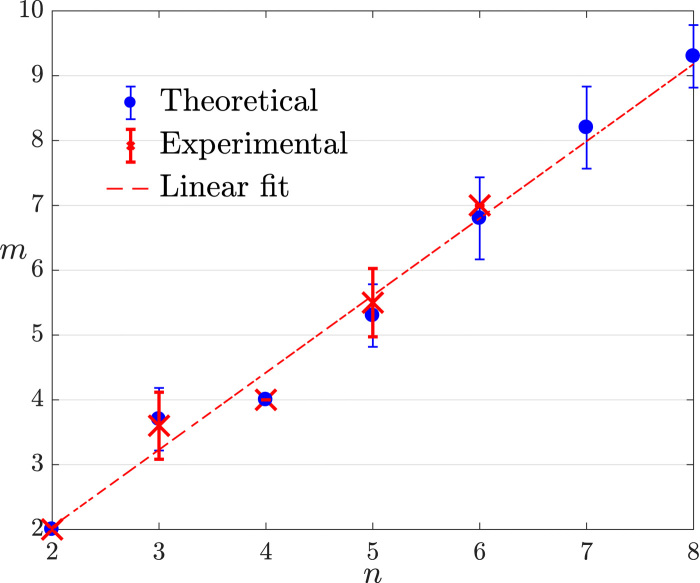
Experimental demonstration of Theorem 1. Scaling of size of the training set *m* required to learn a GHZ state as a function of the number of qubits *n*. Experimental data points (red crosses) are obtained using the experimental setup (II). Each data point is obtained using 50 different, randomly generated sets of measurement configurations drawn from $D_{\left(\text{II}\right)}$ (see Materials and Methods for further details). Error bars show the SD for an average of 10 different runs of the algorithm to estimate *m*. The red line is a linear fit on the experimental data points with equation *m* = 1.19*n* − 0.34. The learning parameters are ε = 0.15, γ = 0.2, and δ = 0.2.

We demonstrate the linear scaling of Theorem 1 over a GHZ of the type described in Eq. 4 and generated by exploiting setup (II). Our algorithm takes as input the error parameters ε, γ, and δ and, for a given *n*, outputs the minimum *m* such that a training set that respects Eq. 1 is generated with probability *p* = 1 − δ. We present the results in Fig. 5 for both numerical and experimental data. The experimental data demonstrate that quantum states are PAC learnable. A linear fit performed on the experimental data returns a slope value of 1.1. This implies that the value of the scaling constant *K* in Eq. 2, left undetermined in Theorem 1, is compatible with learning in an experimental setting. The values obtained from the linear fit in Fig. 5 show that learning a 20-qubit state would require ~ 23 measurements. Notice that a 20-qubit stabilizer state has 1,048,576 stabilizers and that the learning algorithm does not exploit the group structure of the state. In this sense, the algorithm “learns” that the state can be represented using only the generators of the group.

## DISCUSSION

Our work experimentally demonstrates that quantum states that are close to GHZs, as a hypothesis class, are PAC learnable under two nontrivial probability distributions. This result, first proved in (*14*), shows that a number of copies of the state that grows polynomially with the number of qubits is sufficient to PAC-learn the state. This is in marked contrast with the, much stronger, tomographic setting where, for an arbitrary quantum state, the number of copies must grow exponentially. The line of research that seeks to establish how much information is really contained in a quantum state, and thereby to gain insight about the reality of the wave function, has recently found a new addition in the “shadow tomography” protocol proposed by Aaronson (*31*). This protocol can predict the outcomes of *M* different two-outcome measurements on a *D*-dimensional state, to high accuracy, by measuring only poly(log(*D*), log(*M*)) copies of the state. An experimental demonstration of this protocol is a natural future direction and would be a valuable addition to our physical comprehension of these theoretical results.

From a broader perspective, our work constitutes an example of how the techniques developed in the framework of computational learning theory can be used within quantum information. The interplay of these two fields, recently surveyed by Arunachalam and de Wolf (*32*), can offer new tools to investigate properties of quantum states and circuits and can help to identify cases in machine learning where classical and quantum computation behave differently. This is particularly important in light of the recent advances in quantum algorithms for machine learning [recently reviewed by Biamonte *et al*. (*33*) and by Ciliberto *et al*. (*34*)] where, despite the growing interest for the topic, it is still unclear whether caveat-free speedups can be attained [for a critical discussion, see (*34*, *35*)].

## MATERIALS AND METHODS

### The learning theorem

The theorem proved in (*14*) states:

**Theorem 1.** Let ρ be an *n*-qubit state, let D be a distribution over two-outcome measurements, and let ε = (*E*_1_, …, *E*_*m*_) consist of *m* measurements drawn independently from D. Suppose that we are given bits *B* = (*b*_1_, …, *b*_*m*_), where each *b*_*i*_ is 1 with independent probability Tr(*E*_*i*_ρ) and 0 with probability 1 − Tr(*E*_*i*_ρ). Suppose also that we choose a hypothesis state σ to minimize the quadratic functional $f(\sigma )=\sum_{i=1}^{m}{\left(\text{Tr}(E_{i}\sigma )-b_{i}\right)}^{2}$. Then, there exists a positive constant *K* such that

$$\underset{E\in D}{\text{Pr}}[|\text{Tr}(E\sigma )-\text{Tr}(E\rho )|>\gamma ]\leq \varepsilon$$

with a probability of at least 1 − δ over E and *B*, provided that

$$m\geq \frac{K}{\gamma^{4}\varepsilon^{2}}(\frac{n}{\gamma^{4}\varepsilon^{2}}{\text{log}}^{2}\frac{1}{\gamma \varepsilon }+\text{log}\frac{1}{\delta })$$

Here, rather than working with single-measurement outcomes *b*_*i*_, we are concerned with estimated expected values

$$\text{Tr}(E_{i}\rho )\approx \sum_{j=1}^{S}b_{i}^{\left(j\right)}/S$$

where each $b_{i}^{\left(j\right)}$ is the *j*th measurement outcome corresponding to *E*_*i*_. To show that the hypothesis σ generated by considering the expected values is equivalent to that obtained by taking the measurements outcome *b*_*i*_, we define

$$f\prime =\sum_{i=1}^{m\prime }{\left(\text{Tr}(E_{i}\sigma )-\text{Tr}(E_{i}\rho )\right)}^{2}$$

If we take *m* = *m*′*S* and solve for σ, then in the equations *df*/*d*σ = 0 and *df*′/*d*σ = 0, it is possible to verify that the hypothesis that minimizes the function *f*′ is also satisfying *f*.

### The learning distributions

We used different learning distributions for the two experimental setups, $D_{\left(I\right)}$ and $D_{\left(\text{II}\right)}$. The distribution $D_{\left(I\right)}$ is uniform over the set of stabilizer measurements (*36*) of the GHZ state minus the identity matrix. The distribution $D_{\left(\text{II}\right)}$ is uniform over the set of stabilizer measurements in *X* and *Z* of the GHZ state minus the identity matrix. A GHZ state (*20*) is a type of stabilizer state. A stabilizer state |ψ〉 is the unique eigenstate with eigenvalue +1 of a set of *N* commuting multilocal Pauli operators *P*_*i*_s, that is, *P*_*i*_|ψ〉 = |ψ〉, where *P*_*i*_ = ⊗ _*j*_*w*_*j*_ and *w*_*j*_ ∈ {*I*, σ^*x*^, σ^*y*^, σ^*z*^} are the Pauli matrices. We define the *P*_*i*_ as the stabilizers of the state.

There are 2^*n*^ different stabilizers for an *n*-qubit stabilizer state. Because one of the stabilizers is always the identity (whose eigenvalue is 1 for every state), we chose not to include this measurement in those sampled by D.

Each *P*_*i*_ is a two-outcome observable (with eigenvalues +1 or −1). We constructed the POVM elements $E_{i}^{\left(1\right)}$ and $E_{i}^{\left(2\right)}$ of the observable *P*_*i*_ by noting that $E_{i}^{\left(1\right)}+E_{i}^{\left(2\right)}=I$ and $E_{i}^{\left(1\right)}-E_{i}^{\left(2\right)}=P_{i}$. The POVM element $E_{i}^{\left(1\right)}$ can be then written as $E_{i}^{\left(1\right)}=(I+P_{i})/2$.

The set of stabilizers of a state form a group under the operation of matrix multiplication. To represent a state, it is then sufficient to consider the *n* stabilizers that generate this group. For an *n*-qubit state, there are *n* elements in the set of generators.

The high variance around *m* = 4 in Fig. 3 can be explained in the following way: Each data point was obtained by averaging over a number of different configurations sampled from $D_{\left(I\right)}$. It is then likely to sample a configuration that includes two generators and two other stabilizers that can be obtained by the product of the generators. It is easy to see how the information content of such a configuration is less than the one where four independent stabilizers are sampled. This will, in turn, limit the ability of σ to output good predictions and will generate the high variance in the data.

### Numerical simulations

We minimized the function *f* over the positive semidefinite matrices of unit trace, with a variant of the Frank-Wolfe algorithm (*37*) developed by Hazan (*38*). All our simulations were performed using 300 iterations of the Hazan algorithm.

### Experimental details

For the experimental setups of Fig. 2, a pump laser with λ = 397.5 nm was produced by a second harmonic generation process from a Ti:Sapphire mode locked laser with a repetition rate of 76 MHz. Photon pairs entangled in the polarization degree of freedom were generated by exploiting a type II SPDC in 2-mm-thick β-barium borate crystals. The photons generated by SPDC are filtered in wavelength and spatial mode by using narrow band interference filters and SMFs, respectively. After coupling into SMFs, the spatial mode becomes a fundamental Gaussian mode (*TEM*_00_) with a null-associated OAM.

## Supplementary Material

http://advances.sciencemag.org/cgi/content/full/5/3/eaau1946/DC1

Download PDF

Experimental learning of quantum states

## SUPPLEMENTARY MATERIALS

Supplementary material for this article is available at http://advances.sciencemag.org/cgi/content/full/5/3/eaau1946/DC1 Supplementary Appendix A. Theorem 1 with expected measurement values Supplementary Appendix B. Algorithm to estimate the scaling of *m* Supplementary Appendix C. The Hazan’s algorithm
