# Supplementary material for: Experimental learning of quantum states
Source: Sci Adv. 2019 Mar 29;5(3):eaau1946. doi: 10.1126/sciadv.aau1946 (PMC6440753; doi:10.1126/sciadv.aau1946)
Supplement: http://advances.sciencemag.org/cgi/content/full/5/3/eaau1946/DC1 [file supp_5_3_eaau1946__index.html]

Science Advances | Science AdvancesAAASSearchScience AdvancesMenu

## Supplementary Materials

**This PDF file includes:**

- Supplementary Appendix A. Theorem 1 with expected measurement values
- Supplementary Appendix B. Algorithm to estimate the scaling of *m*
- Supplementary Appendix C. The Hazan’s algorithm

Download PDF

**Files in this Data Supplement:**

- Adobe PDF - aau1946\_SM.pdf
