# Supplementary material for: Experimental learning of quantum states
Source: Sci Adv. 2019 Mar 29;5(3):eaau1946. doi: 10.1126/sciadv.aau1946 (PMC6440753; doi:10.1126/sciadv.aau1946)
Supplement: Download PDF [file aau1946_SM.pdf]

Science Advances
AAAS

advances.sciencemag.org/cgi/content/full/5/3/eaau1946/DC1

Supplementary Materials for

Experimental learning of quantum states

Andrea Rocchetto*, Scott Aaronson, Simone Severini, Gonzalo Carvacho, Davide Poderini, Iris Agresti, Marco Bentivegna, Fabio Sciarrino*

*Corresponding author. Email: andrea.rocchetto@spc.ox.ac.uk (A.R.); fabio.sciarrino@uniroma1.it (F.S.)

Published 29 March 2019, *Sci. Adv.* **5**, eaau1946 (2019)
DOI: 10.1126/sciadv.aau1946

This PDF file includes:

Supplementary Appendix A. Theorem 1 with expected measurement values
Supplementary Appendix B. Algorithm to estimate the scaling of *m*
Supplementary Appendix C. The Hazan's algorithm

Supplementary Appendix A. Theorem 1 with expected measurement values

Theorem 1 is stated in terms of the single measurement outcomes b_i. Here we show that the results of the theorem still hold if, rather than consider single measurement outcomes, we work with the estimated expected values of $\mathrm{Tr}\,(E_i\rho) \approx \sum_{j=1}^{s} b_i^{(j)}/s$ where each $b_i^{(j)}$ is 1 with independent probability $\mathrm{Tr}\,(E_i\rho)$ and 0 with probability $1 - \mathrm{Tr}\,(E_i\rho)$. To establish the equivalence it suffices to show that the σ that minimises $f = \sum_{i=1}^{m}(\mathrm{Tr}(E_i\sigma) - b_i)^2$ also minimises $f' = \sum_{i=1}^{m'} \left(\mathrm{Tr}\,(E_i\sigma) - \mathrm{Tr}\,(E_i\rho)\right)^2$ where $m = m's$.

For an integer s, let $[s]$ denotes the set $\{1, \ldots, s\}$. If we assume that there exist s different measurements $\{b_i^{(j)}\}_{j\in[s]}$ of each operator E_i we can rewrite f by grouping together measurement outcomes that correspond to a single POVM

$$\begin{aligned}
\sum_{i=1}^{m}(\mathrm{Tr}(E_i\sigma) - b_i)^2 &= (\mathrm{Tr}(E_1\sigma) - b_1^{(1)})^2 + (\mathrm{Tr}(E_1\sigma) - b_1^{(2)})^2 + \cdots + (\mathrm{Tr}(E_{m'}\sigma) - b_{m'}^{(s-1)})^2 + (\mathrm{Tr}(E_{m'}\sigma) - b_{m'}^{(s)})^2 \\
&= \sum_{i=1}^{m'}\left[s\,(\mathrm{Tr}(E_i\sigma))^2 + \sum_{j=1}^{s}(b_i^{(j)})^2 - 2\,\mathrm{Tr}(E_i\sigma)\sum_{j=1}^{s} b_i^{(j)} \right] \\
&= \sum_{i=1}^{m'} s\left[(\mathrm{Tr}(E_i\sigma))^2 + \sum_{j=1}^{s}(b_i^{(j)})^2/s - 2\,\mathrm{Tr}(E_i\sigma)\sum_{j=1}^{s} b_i^{(j)}/s \right]
\end{aligned}$$

Equivalently f' can be expressed as

$$\begin{aligned}
\sum_{i=1}^{m'}\left(\mathrm{Tr}\,(E_i\sigma) - \mathrm{Tr}\,(E_i\rho)\right)^2 &= \sum_{i=1}^{m'}\left(\mathrm{Tr}\,(E_i\sigma) - \sum_{j=1}^{s} b_i^{(j)}/s\right)^2 \\
&= \sum_{i=1}^{m'}\left[\left(\mathrm{Tr}\,(E_i\sigma)\right)^2 + \left(\sum_{j=1}^{s} b_i^{(j)}/s\right)^2 - 2\,\mathrm{Tr}\,(E_i\sigma)\sum_{j=1}^{s} b_i^{(j)}/s \right]
\end{aligned}$$

The minimum of $f(\sigma)$ is found for

$$\frac{df(\sigma)}{d\sigma} = \sum_{i=1}^{m'}\left[\frac{d\,\mathrm{Tr}(E_i\sigma)^2}{d\sigma} - 2\frac{d\,\mathrm{Tr}(E_i\sigma)}{d\sigma}\sum_{j=1}^{s} b_i^{(j)}/s \right] = 0$$

Equivalently, we get for f'

$$\frac{df'(\sigma)}{d\sigma} = \sum_{i=1}^{m'}\left[\frac{d\,\mathrm{Tr}\,(E_i\sigma)^2}{d\sigma} - 2\frac{d\,\mathrm{Tr}\,(E_i\sigma)}{d\sigma}\sum_{j=1}^{s} b_i^{(j)}/s \right] = 0$$

It is easy to see how f and f' are minimised by the same σ.

Supplementary Appendix B. Algorithm to estimate the scaling of m

With algorithm 1 we estimate the minimum number of measurements m that allows us to PAC-learn ρ with accuracy parameters ϵ, γ and success probability $1 - \delta$. For each iteration of i the algorithm generates a set of measurements drawn from either $\mathcal{D}_{(I)}$ or $\mathcal{D}_{(II)}$. We give the pseudocode for the case of $\mathcal{D}_{(I)}$. The support of $\mathcal{D}_{(I)}$ is the set $\mathcal{V}$ of stabiliser measurements of the state minus the identity operator. Because each stabiliser state has 2^n stabiliser measurements we have $|\mathcal{V}| = 2^n - 1$.

The case for $\mathcal{D}_{(II)}$ is identical apart for the support of $\mathcal{D}_{(II)}$ that is now the set $\mathcal{W}$ of the stabiliser measurements on X and Z of the state minus the identity operator.

Algorithm 1 Find minimum m that allows to PAC-learn ρ

Input: quantum state ρ, number of qubits n, distribution $\mathcal{D}_{(I)}$, error parameters ϵ, γ, δ, number of different training sets used for the estimate i_{MAX}
Output: minimum value of m that satisfies the conditions of Theorem 1

$m = 1$
repeat
  $\delta_{est} = 0$
  for $i = 1 \dots i_{MAX}$ **do**
    Generate training set $T = \{(E_i, \text{Tr}(E_i\rho))\}_{i\in[m]}$ with random measurements drawn from $\mathcal{D}_{(I)}$
    $\sigma = \text{HAZAN}(T, n)$
    for every $E \in \mathcal{V}$ **do**
      if $|\text{Tr}(E\sigma) - \text{Tr}(E_i\rho)| > \gamma$ **then**
        $\epsilon_{\text{est}} += 1/|\mathcal{V}|$
      end if
    end for
    if $\epsilon_{\text{est}} > \epsilon$ **then**
      $\delta_{est} += 1/i_{\text{MAX}}$
    end if
  end for
  $m = m + 1$
until $\delta_{est} < \delta$

Supplementary Appendix C. The Hazan's algorithm

As discussed the problem of learning quantum states can be cast as a convex program. In the formulation given in Eq. 3 the goal is to minimise the objective function $f(\sigma) = \sum_{i=1}^{m}(\text{Tr}(E_i\sigma) - \text{Tr}(E_i\rho))^2$ over the positive semidefinite matrices of unit trace. Because both the space of positive semidefinite matrices of unit trace and the objective function are convex, we are dealing with a constrained convex optimisation problem. A polynomial time algorithm for this class of problems is the Frank-Wolfe algorithm [38] for optimising a single function over the bounded positive semidefinite cone. In our simulations we use an extension of this work, developed by Elad Hazan [39], specifically designed for learning quantum states with the procedure described in Theorem 1.

Algorithm 2 Hazan's algorithm

Input: training set $T = \{(E_i, \text{Tr}(E_i\rho))\}_{i\in[m]}$, Hilbert space dimension $N = 2^n$, and maximum number of iterations k_{MAX}
Output: hypothesis state σ

1: Initialise $\sigma_0 = I/N$
2: **for** $k = 1$ **to** k_{MAX} **do**
3:   **begin**
4:   Compute the smallest eigenvector v_k of $\nabla f(\sigma_k)$
5:   Let $\alpha = \frac{1}{k}$
6:   Update $\sigma_{k+1} = \sigma_k + \alpha_k(v_k v_k^T - \sigma_k)$
7:   **end**

We can compute analytically step 4 by using that $\frac{\partial \text{Tr}(F(\mathbf{X}))}{\partial \mathbf{X}} = f(\mathbf{X})^T$, where f is the scalar derivative of F, and the hermiticity of the measurement operators E_i

$$\begin{aligned}
\nabla f(\sigma_k) &= \frac{\partial f(\sigma_k)}{\partial \sigma_k} \\
&= 2\sum_{i=1}^{m}(\text{Tr}(E_i\sigma_k) - \text{Tr}(E_i\rho))E_i^T \\
&= 2\sum_{i=1}^{m}(\text{Tr}(E_i\sigma_k) - \text{Tr}(E_i\rho))E_i
\end{aligned}$$
